# Gallstone Ileus: An Improbable Cause of Mechanical Small Bowel Obstruction

**DOI:** 10.7759/cureus.11460

**Published:** 2020-11-12

**Authors:** Sara Lourenço, Ana Marta Pereira, Jose Reis, Marta Guimarães, Mário Nora

**Affiliations:** 1 General Surgery, Centro Hospitalar de Entre Douro e Vouga, Santa Maria da Feira, PRT

**Keywords:** gallstone ileus, small bowel obstruction

## Abstract

Gallstone ileus (GI) is a rare complication of cholelithiasis and a rare cause of small bowel obstruction. It usually affects elderly women and the symptoms are nonspecific, both contributing to a delay in diagnosis and a high mortality rate. It is necessary to have a high suspicion index for diagnosis and abdominal CT is the gold standard imaging for the diagnosis.

We present a case report of an 87-year-old man who presented to the ED with abdominal pain and vomiting for the last 20 days. A GI was diagnosed and he underwent enterolithotomy to remove the stone. Unfortunately, the patient died on the 13th postoperative day with multiorgan failure.

The treatment and the time at which it is performed must be adapted to each patient.

## Introduction

Gallstone ileus (GI) is a rare complication of cholelithiasis and is defined as a mechanical intestinal obstruction secondary to the presence of a gallstone in the intestinal lumen [1]. It accounts for 1%-3% of intestinal obstruction cases and is usually diagnosed in the older female population [2]. Signs and symptoms are nonspecific [3] and diagnosis is made after an imaging exam. Because it is a rare entity and the affected patients are old, it is necessary to have a high suspicion index for diagnosing GI [4].

We present a case of a GI in a patient with no previous biliary history, who underwent an urgent laparotomy.

## Case presentation

An 87-year-old man presented to the ED with diffuse abdominal pain and vomiting for the last 20 days, associated with weight loss, anorexia, and sporadic diarrhea. He denied fever, urinary symptoms, or similar previous episodes. He was prescribed with metoclopramide and simethicone without improvement.

In his past medical history there is reference to arterial hypertension and dyslipidemia, treated with olmesartan, sinvastatine, and ezetimibe. In his surgical history there was a colon surgery 30 years ago for colon malignancy. This surgery was complicated by anastomosis dehiscence with stoma confection, and after that the intestinal transit was re-established.

On physical examination, the patient was alert and oriented, vitals were within normal limits, with absence of icterus or jaundice. The abdomen was soft, tympanic with moderate epigastric tenderness without any lump or organomegaly. As per rectal examination, the rectal vault did not reveal any fecal impaction and hernia points were free.

The blood analysis revealed a hyponatremia (135 mmol/L), hypokalaemia (3.4 mmol/L), acute kidney injury (creatinine 1.5 mg/dL), and increased bilirubin (bilirubin 1.86 mg/dL) with a moderate increase in C reactive protein (41 mg/L), despite normal white blood cells count.

**Figure 1:**
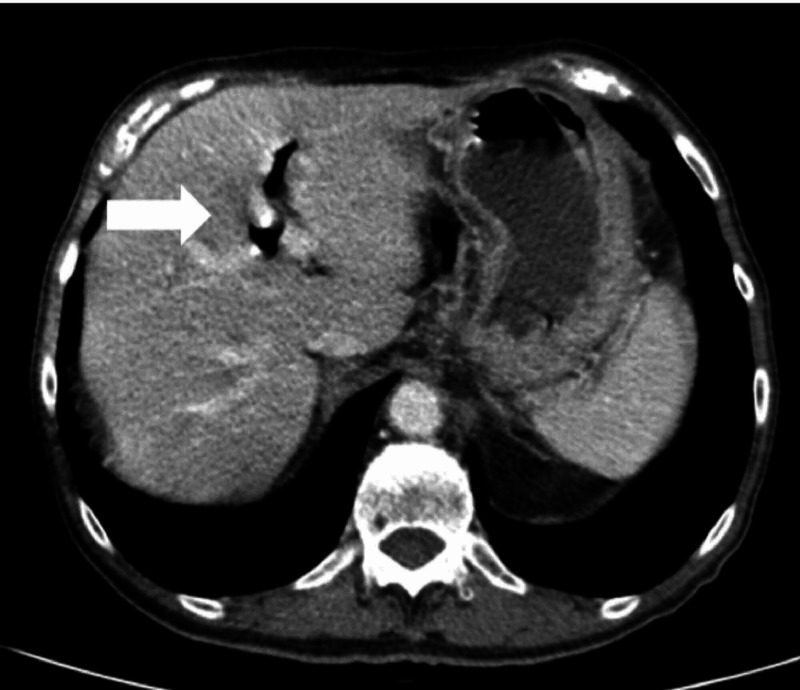
Abdomino-pelvic CT: gastric distension and aerobilia (white arrow).

**Figure 2:**
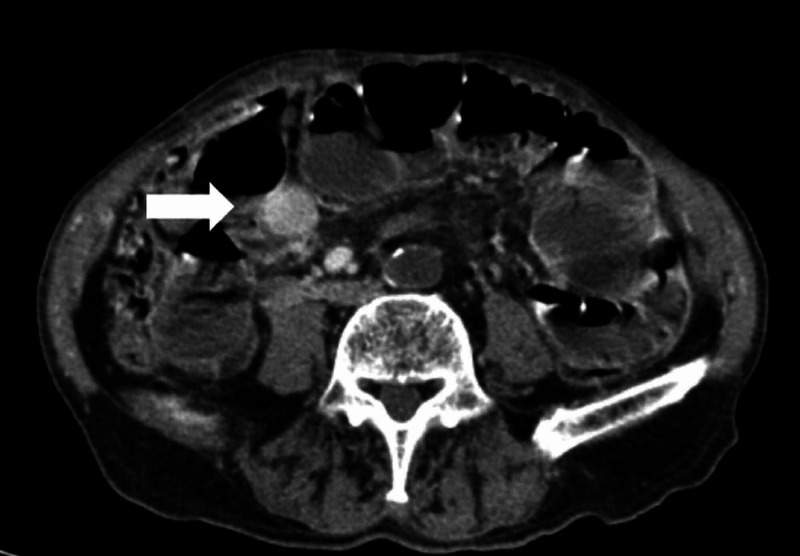
Abdomino-pelvic CT (axial section) revealing the gallstone (white arrow) impacted in terminal ileum, causing proximal small bowel dilatation.

**Figure 3:**
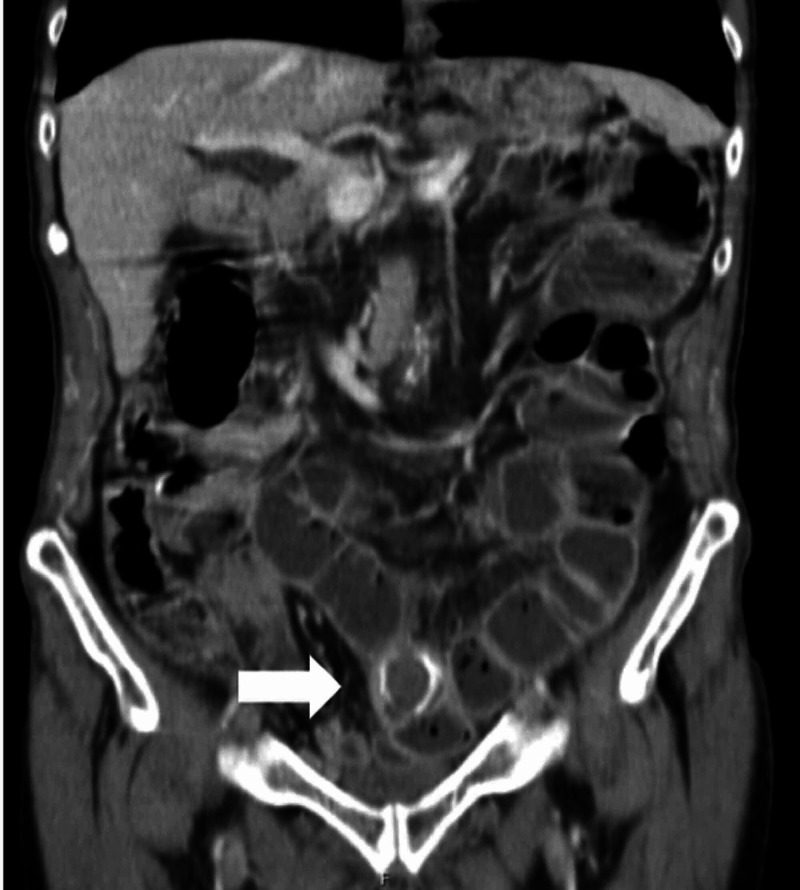
Abdomino-pelvic CT (coronal section) revealing the gallstone (white arrow) impacted in terminal ileum, causing proximal small bowel dilatation.

The abdomino-pelvic CT revealed intra-hepatic biliary ducts dilatation with aerobilia, associated with air in the gallbladder (Figure 1). Small bowel dilatation with loops thickening and an ovoid mass with 3 cm at distal ileus, with thinned and reduced caliper of intestinal loops distally to this structure, suggested a small bowel obstruction by a gallstone that migrated from the gallbladder (Figures 2-3).

**Figure 4:**
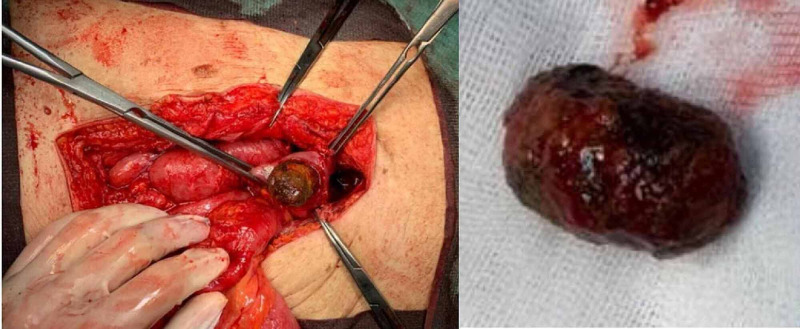
Laparotomy with enterolithotomy to remove the gallstone.

A laparotomy was performed, with laborious adhesiolysis and enterolithotomy to remove the stone (Figure 4).

On the third postoperative day, the patient had a fecaloid drainage by the surgical wound, so a laparotomy was performed. There was generalized peritonitis caused by spotty suture dehiscence that was sutured and a drainage tube was placed. The patient was admitted to the ICU and had biliary drainage without signs of peritoneal irritation. On the 10th postoperative day, the clinical condition deteriorated, which necessitated a workup. Abdominal CT revealed free liquid and two abscesses in the Morrison’s pouch and right iliac fossa with 38 mm x 17 mm and 80 mm x 30 mm, respectively. The patient was hemodynamically stabilized and the surgical team revised the laparotomy, which revealed complete dehiscence of the small bowel suture and the two abscesses. The dehiscence was sutured and the abscesses were drained. Two drainage tubes were placed, at dehiscence level and at the right iliac fossa. The patient was admitted to the ICU and died after three days with multiorgan failure.

The pathological examination revealed a cholesterol gallstone with 4 cm x 2 cm.

## Discussion

The GI is a cause of mechanical bowel obstruction due a biliary calculus. The first case of GI was described by Thomas Bartholin in 1654, in an autopsy study [5].

This occurs due to chronic pressure necrosis from large stones in an inflamed gallbladder, forming a cholecystoenteric fistula, allowing gallstones direct access to the gut. Usually the fistula is located between the gallbladder and duodenum (first and second part), but fistulas to the stomach and colon have been described [6]. The majority of the gallstones smaller than 2-2.5 cm may pass spontaneously through a normal gastrointestinal tract. However, stones larger than 5 cm are more likely to be impacted [3]. The most common impaction site of the gallstone is the ileum (50%-60.5%), jejunum (3.5%-14.6%), and the colon (3%-4.1%) [7].

It represents 1%-3% of mechanical small bowel obstruction. It is more common in patients over the age of 65 representing up to 25% of small bowel obstruction, and in this age group it is more frequent in females (3.5-6:1) [8].

Clinical symptoms differ, depending on the site of obstruction. In cases of bowel obstruction, vomiting, constipation and abdominal distension and pain are prevalent. There are three clinical types of this disease: acute (‘classic’ GI), sub-acute (bowel sub-occlusion), and chronic (Karewsky syndrome - repeated episodes of pain due to the passage of the gallstone into the bowel) [9]. The physical examination and laboratory tests do not point to a particular cause of bowel obstruction; however, electrolyte imbalances are frequent.

Imaging is the key for the diagnosis of GI. In 1941, Rigler described the triad of radiological signs for GI on plain film. The Rigler’s triad includes the presence of a radiopaque stone (presenting in fewer than 10% of the cases), pneumobilia (Gotta-Mentschler sign), and bowel loop distention. The presence of at least two of the three signs is diagnostic. The evidence in a second plain film of a change in stone position is known as Rigler’s tetrad.

Furthermore, it was described the fifth radiological sign consisting of two adjacent small air-fluid levels in the right upper quadrant, the median collection being in the duodenal bulb and the lateral in the gallbladder [10].

However, the contrast-enhanced CT is considered the best method for GI diagnosis. This is confirmed by a retrospective study, developed by Lassandro et al., revealing that Rigler’s triad is present in 14.8% on plain abdominal films, 11.11% in abdominal ultrasonography, and 77.78% in abdominal CT [11]. Abdominal CT provides important information regarding the number, size, and location of the stones and of intestinal obstruction or direct visualization of a biliary-enteric fistula and helps clinicians in the therapeutic management [12].

The diagnosis of GI is not always straightforward and requires a high index of suspicion [13]. The differential diagnosis includes other causes of mechanical small bowel obstruction, such as postoperative adhesions, hernias, small bowel tumors, and foreign bodies.

There is no consensus regarding therapeutic timing and surgical procedure; however, the main objective in treating GI is to relieve the intestinal obstruction, remove the stone, and if possible, avoid future episodes [3].

The GI, before a surgical condition, is a systemic disease. Patients frequently have severe imbalances, such as fluid, electrolyte and metabolic disorders that exacerbate comorbidities. So, an adequate and timely treatment is based on the clinical stability of the patient. The treatment of GI is, therefore, a combination of medical and surgical treatment [14].

The surgical approach may be enterolithotomy alone and enterolithotomy, cholecystectomy, and fistula closure in one-stage or two-stage procedure. Enterolithotomy alone is the minimum surgery that can relieve the obstruction in an emergency situation. It is safe in both low- and high-risk patients and requires a shorter operating time as it is technically less demanding than the one-stage procedure [15-16]. The disadvantage of enterolithotomy alone is recurrent biliary symptoms that occur in 10% of the patients and an increased risk of cholangiocarcinoma [10, 17]. The two-stage surgical procedure has been suggested in young patients at risk of biliary complications. The time-lapse between the first stage and the second stage ranges from four to six weeks after the first surgery [18]. The resection of an intestinal segment is indicated when there is an irreversible vascular compromise or bowel perforation [19].

Although the experience in minimally invasive surgical approach is developing, adequate management of low-risk patients has allowed successful results. Laparoscopy is used only in 10% of surgically managed GI, with a high conversion rate (53.3%) [3].

Considering that most patients with GI often are older and high-risk candidates for surgery, endoscopic management (or extracorporeal shock wave lithotripsy) should be considered as an alternative approach [3].

Gallstone ileus is associated with significant morbidity and mortality. There are, at least, four main reasons that might be responsible for the high number of lethal cases. First of all, GI is a disease of the elderly. Second, concomitant diseases, such as cardiorespiratory diseases and/or diabetes mellitus, are frequent. Third, uncommon symptoms may delay the diagnosis. Fourth, the postoperative recovery is also hampered because age-related complications such as pneumonia or cardiac failure are more frequent than surgery-associated complications [20].

## Conclusions

The GI is a rare condition causing intestinal occlusion. It occurs predominantly in the elderly and is associated with significant mortality. It is necessary to have a high index of suspicion for diagnosing GI. The treatment and the time at which it is performed must be adapted to each patient.
